# State of the Art on Inherited Retinal Dystrophies: Management and Molecular Genetics

**DOI:** 10.3390/jcm14103526

**Published:** 2025-05-18

**Authors:** Marcella Nebbioso, Marco Artico, Magda Gharbiya, Alice Mannocci, Paolo Giuseppe Limoli, Danilo Iannetta, Luigi Donato

**Affiliations:** 1 Department of Sense Organs, Faculty of Medicine and Odontology, Sapienza University of Rome, P.le A. Moro 5, 00185 Rome, Italy; marco.artico@uniroma1.it (M.A.); magda.gharbiya@uniroma1.it (M.G.); danilo.iannetta@uniroma1.it (D.I.); 2 Department for the Promotion of Human Sciences and Quality of Life, University San Raffaele, 00166 Rome, Italy; alice.mannocci@uniroma5.it; 3 Clinical and Molecular Epidemiology, IRCCS San Raffaele, 00163 Rome, Italy; 4 Low Vision Research Centre of Milan, p.zza Sempione 3, 20145 Milan, Italy; paololimoli@libero.it; 5 Department of Biomedical and Dental Sciences and Morphofunctional Imaging, Division of Medical Biotechnologies and Preventive Medicine, University of Messina, 98125 Messina, Italy; luigi.donato@unime.it; 6 Department of Biomolecular Strategies, Genetics and Cutting-Edge Therapies, Istituto Euro Mediterraneo di Scienza e Tecnologia (I.E.ME.S.T.), 90139 Palermo, Italy

**Keywords:** gene therapy, genetic counseling, genetic mutations, genome editing, inherited IRDs, inherited retinal diseases, retinitis pigmentosa, sequencing, syndromic inherited retinal dystrophy

## Abstract

Inherited retinal dystrophies (IRDs) represent a group of heterogeneous disorders caused by gene mutations primarily affecting retinal photoreceptors. In addition to vision loss, other symptoms may lead to visual impairment, such as altered visual fields, hemeralopia, glare sensitivity, and impaired color vision. These conditions almost always complicate with the onset of cataracts, macular edema or atrophy, glaucoma, etc. A brief overview of key genes involved in the most common and well-known IRDs is provided, followed by clinical and diagnostic implications. The study of IRDs has seen a significant acceleration in recent decades, owing to advances in molecular genetics with the introduction of exome sequencing (WES) and genome-wide association studies (GWASs), which have facilitated the identification of a broad spectrum of genes associated with IRDs. This has led to the classification of five genetic variants, based on the criteria of the American College of Medical Genetics and Genomics (ACMG), serving as a guide for interpreting genetic reports. Next, approaches to genomic editing therapies and research directions regarding artificial intelligence (AI) and machine learning (ML) are discussed. The paper concludes with an examination of the inevitable ethical and regulatory issues, typically driven by regulatory bodies such as the Food and Drug Administration (FDA).

## 1. Introduction

Inherited retinal dystrophies (IRDs) are a complex group of heterogeneous genetic disorders caused by specific gene mutations that primarily affect the chorioretinal cells, particularly the photoreceptors. These diseases are generally severe and can lead to varying degrees of visual impairment, potentially resulting in legal blindness and sometimes being associated with optic nerve subatrophy or atrophy, or being part of complex syndromic presentations. Among the most common symptoms, which may appear from early childhood or adulthood depending on the type of disease, the following are primarily observed: hemeralopia, nyctalopia, reduced peripheral visual field, central scotoma, glare sensitivity, and altered color perception [1,2,3]. Possible complications include early onset of bilateral cataracts, chronic glaucoma, cystoid macular retinal edema, central chorioretinal atrophy, and macular choroidal neovascularization. Recent studies have identified over 300 genes associated with various forms of IRDs, demonstrating a complex genetic landscape where single mutations can lead to different phenotypes and similar clinical manifestations can arise from mutations in different genes. Therefore, the term IRDs is more commonly used, sometimes without identifying a precise phenotype [2,3,4]. The study of IRDs has undergone significant acceleration in recent decades, driven by advancements in molecular genetics. Initially limited to phenotypic observations and pedigree analysis, research has experienced a paradigmatic shift with the introduction of whole exome sequencing (WES) and genome-wide association studies (GWASs), enabling the identification of a broad spectrum of genes linked to IRDs. Such discoveries have not only enhanced diagnostic accuracy but have also opened new frontiers in the treatment of these conditions, with the development of targeted genetic tests and the introduction of innovative therapies aimed at modulating or reversing disease progression [2,3,4,5,6].

In this heterogeneous and at times varied group of complex visual disorders, mutations in crucial genes cause malfunctioning of retinal photoreceptors; the alteration subsequently affects the biological mechanism that converts light into bioelectrical signals, enabling transmission along the visual pathways to the brain, enabling accurate sensory processing [7,8].

**Figure 1:**
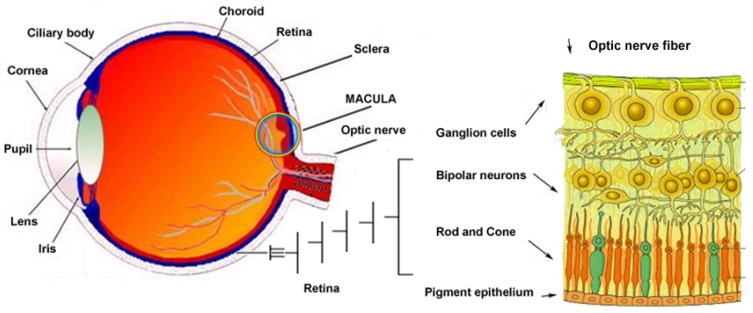
Ocular anatomy and retinal cell structure.

Mutations that impact retinal cells disrupt the normal physiological process (Figure 1), leading to the manifestation of symptoms specific to IRDs. Among the possible molecular mechanisms of these mutations are the following [8,9,10]:

1. Loss of function, when the gene product is either not produced or is nonfunctional;
2. Gain of function, where a change in the gene product becomes toxic to the cell;
3. Dominant-negative effect, when the mutant gene product interferes with the normal function of the protein.

For instance, mutations in the *RPE65* gene disrupt the visual cycle, causing the accumulation of toxic byproducts and photoreceptor cell death, while mutations in the *ABCA4* gene lead to defective retinaldehyde transport, causing similar accumulations and subsequent cell death. Advancements in genetic technologies, such as next-generation sequencing (NGS), have revolutionized diagnostic capabilities, enabling comprehensive screening of all known IRD-associated genes in a single test [10,11,12,13]. This not only improves diagnostic accuracy but also helps personalize management strategies. An interdisciplinary understanding of the genetic basis of IRDs is considered crucial by the international scientific community for the development of advanced diagnostic, management, therapeutic, and research strategies, which will significantly improve the outcomes for patients with visual disabilities, facilitating a proper approach to the well-being of individuals and their social and educational integration [4,5,6,7]. Therefore, the present study aims to explore, as comprehensively as possible, the genetic and diagnostic framework of a group of tissue disorders characterized by progressive visual function loss due to retinal degeneration, responsible for civil disability or legal blindness.

## 2. Brief Overview of the Key Genes Involved in IRDs (Figure 2 and Figure 3)

### 2.1. Leber Congenital Amaurosis (LCA)

The LCA represents the most severe form within the spectrum of IRDs, as it is characterized by an early onset and rapid progression to blindness. Genetic mutations in *RPE65*, *GUCY2D*, *CRB1*, and *CEP290* are among the most common causes of LCA, with each gene contributing uniquely to the pathology [11,14,15,16]. *RPE65* plays a central role in the visual cycle, and its mutations disrupt the recycling of retinoids necessary for phototransduction. Children with *RPE65* mutations typically present in the first few months of life with severe visual problems, nystagmus, and an absent or severely reduced electroretinogram (ERG). Gene therapy targeted at the *RPE65* mutation has made significant strides, with Luxturna (voretigene neparvovec) becoming the first Food and Drug Administration (FDA)-approved gene therapy for a hereditary disease [16,17,18].

**Figure 2:**
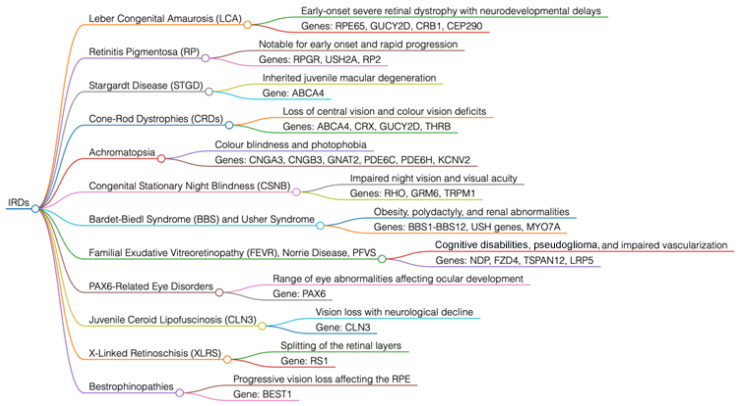
Main forms of inherited retinal dystrophies (IRDs) and their causative genes. The dendrogram summarizes the main forms of IRDs, with a brief description of the clinical phenotype associated with alterations in the main causative genes.

**Figure 3:**
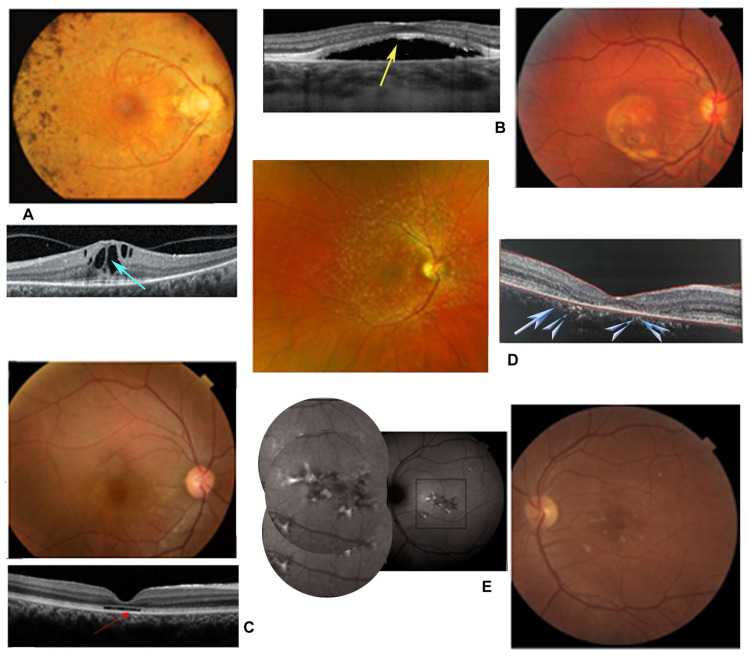
Fundus images of patients with inherited retinal dystrophies (IRDs). (**A**) Retinitis pigmentosa with bone spicule-like pigment accumulations in the intermediate region of the retinal periphery. Optical coherence tomography (OCT) examination shows optically empty cysts due to the presence of cystoid macular edema (blue arrow). (**B**) Best disease with yellowish lipofuscin deposits, “egg yolk” type, in the macula. Evident serous detachment of the neuroretina on OCT examination (yellow arrow). (**C**) The fundus image appears normal, but the OCT scan shows delamination of the outer retina due to cone dystrophy (red arrow). (**D**) Stargardt’s disease with fundus flavimaculatus for yellowish pisciform lesions on the posterior pole. Marked thinning of the macular outer retina due to atrophy visible on OCT examination (celestial arrows). (**E**) Butterfly-type bestrophinopathy due to remodeling of the retinal pigmented epithelium in the macular area, more evident in the autofluorescence image (inset).

*GUCY2D*, on the other hand, encodes guanylate cyclase-1, a key enzyme in photoreceptor cells. Mutations in this gene lead to reduced enzyme activity, impairing the recovery phase of phototransduction and causing photoreceptor cell death. Clinically, this manifests as decreased visual acuity and progressive vision loss, often first noted by parents as a lack of visual attention in infancy. *CRB1* mutations are associated with altered development of the outer limiting membrane of the retina, which is crucial for the alignment and function of photoreceptors. Children with *CRB1* mutations may present a range of symptoms, from severe visual impairment to more moderate forms, often with associated retinal dystrophy observed through ocular imaging [16,17,18].

Finally, *CEP290* is involved in ciliary transport processes, and mutations in this gene can lead to a spectrum of LCA with associated extraocular manifestations, including delays in neurological development. The phenotypic and syndromic variability of LCA associated with *CEP290* is broad, with some patients presenting additional renal or neurological involvement, known as Joubert syndrome and Senior–Løken syndrome [16,17].

Currently, in the case of LCA, early genetic diagnosis can radically alter the disease course, paving the way for specific therapies, family planning, and specialized support services to maximize the child’s developmental potential [15,16,17].

### 2.2. Retinitis Pigmentosa (RP)

Retinitis pigmentosa (RP) is characterized by the progressive loss of photoreceptors, leading to hemeralopia, loss of peripheral vision, commonly referred to as tunnel vision, and often associated with central vision impairment [19,20,21]. Mutations in genes such as *RPGR*, *USH2A*, and *RP2* are implicated in both X-linked and autosomal forms of RP. *RPGR* is a critical gene for the development and maintenance of photoreceptor cells [20,21,22,23]. Mutations in this gene, particularly in the ORF15 region, result in the disruption of protein function, compromising the viability of photoreceptors. Patients with *RPGR* mutations typically exhibit symptoms in early childhood, progressing to legal blindness by adulthood [21,22,23]. *USH2A* mutations are more commonly associated with Usher syndrome type II, which presents as RP combined with neurosensory hearing loss. This requires a multidisciplinary approach to management, including visual aids and cochlear implants [19,20,21,22,23]. *RP2* mutations contribute to a smaller subset of X-linked RP, with a clinical presentation similar to that of *RPGR* mutations. However, the onset and severity can vary significantly, even within members of the same family [20,21,22,23]. Recent clinical trials on gene therapy using AAV5-RPGR and AGTC-501 as AAV vectors to deliver functional copies of the RPGR gene, have demonstrated improvements in retinal sensitivity and visual function.

### 2.3. X-Linked Retinoschisis (XLRS)

XLRS, inherited from the mother to male offspring, is a retinal disease caused by mutations in the *RS1* gene, leading to the separation of the inner and/or outer retinal layers, resulting in reduced vision. The RS1 protein is essential for the integrity and adhesion of retinal cells. The hallmark of XLRS on examination is the presence of cystic, spoke-like alterations in the macula, often referred to as “wheel” or “bicycle”-shaped lesions. Patients often present with difficulty reading up close and reduced visual acuity for distance during childhood [24,25,26].

Current therapeutic options are limited, and some patients require surgical intervention for complications such as retinal detachment (RD), particularly if ruptures occur at the areas of peripheral retinoschisis, usually in the temporal retina, where both inner and outer retinal layers separate. Gene therapy studies are ongoing, offering the possibility of direct treatment for the underlying genetic defect [24,25,26].

### 2.4. Stargardt Disease (STGD)

STGD represents the most common form of juvenile macular IRD, primarily associated with mutations in the *ABCA4* gene [27,28]. However, mutations in other genes, such as *ELOVL4* and *PROM1*, are also implicated in variants of the disease. These genetic mutations lead to photoreceptor dysfunction, mainly through the accumulation of lipofuscin in the retinal pigment epithelium (RPE) at the macular site, resulting in progressive loss of central vision. The *ABCA4* protein acts as a transporter in photoreceptor cells and plays a crucial role in the visual cycle by moving retinal derivatives across the cell membrane [28,29,30]. STGD can range from macular dystrophy to cone-rod dystrophy; the clinical course is highly variable and difficult to predict.

Clinically, STGD presents in late childhood or early adolescence with symptoms related to visual deficits, photophobia, and difficulty adjusting to changes in light. Genetic testing for *ABCA4* mutations confirms the diagnosis and aids in prognosis and family planning [28].

The *ELOVL4* gene encodes an enzyme involved in the biosynthesis of very long-chain fatty acids, which are essential for the normal function of retinal photoreceptors. Pathogenic mutations in *ELOVL4* can lead to an autosomal dominant (AD) form of the disease, known as Stargardt-3 and characterized by the production of a truncated protein that negatively interacts with the normal ELOVL4 protein, interfering with its normal function [29,30].

The *PROM1* gene encodes prominin-1, a membrane associated protein studied for its role in maintaining the structural integrity of photoreceptors [27,28,29,30].

### 2.5. Cone and Rod Dystrophies (CRDs)

CRDs are characterized by the primary loss of cones, followed by degeneration of rods. The associated genes, including *ABCA4*, *CRX*, *GUCY2D*, and *THRB*, highlight the diversity of pathways leading to CRDs [31,32,33,34]. *CRX* encodes a transcription factor essential for the development and maintenance of photoreceptor cells. Mutations in *CRX* lead to a range of retinal disorders, including CRDs, where the initial loss of color vision and visual acuity is indicative of cone involvement. As the disease progresses, patients experience night blindness and loss of peripheral vision, symptoms of rod degeneration that occur at a later stage [32,33,34].

Mutations in *GUCY2D*, as seen in LCA, affect the photoreceptor response to light, and in CRDs, patients often progress to significant loss of central vision and deficits in color vision. *THRB*, which encodes the thyroid hormone beta receptor, has been linked to enhanced S-cone syndrome, a form of CRDs where there is increased function of S-cones (blue cones) at the expense of other photoreceptors [32,33,34].

### 2.6. Achromatopsia

Achromatopsia is an autosomal recessive (AR) ocular disorder characterized by a complete lack of color perception, photophobia, severely reduced visual acuity, and, consequently, nystagmus. The condition is associated with mutations in several genes, including *CNGA3*, *CNGB3*, *GNAT2*, *PDE6C*, *PDE6H*, and *KCNV2*, which play essential roles in the cone phototransduction pathway. *CNGA3* and *CNGB3* encode the alpha and beta subunits of the cone cyclic nucleotide-gated channel, essential for converting visual signals into electrical impulses. Mutations in these genes disrupt ion flow, leading to cone dysfunction and the clinical symptoms of achromatopsia [35,36].

Mutations in *GNAT2* affect the alpha subunit of the cone transducing, while *PDE6C* and *PDE6H* involve the catalytic and inhibitory subunits of the cone phosphodiesterase. Mutations in *KCNV2* are responsible for dysfunction of the voltage-gated potassium channel, further contributing to altered cone phototransduction.

Support options for achromatopsia are limited to the use of lenses with photo-selective filters to manage photophobia and low vision aids to improve vision. Gene therapy studies focused on restoring cone function through targeted gene delivery are ongoing, offering a promising outlook for individuals affected by this condition [35,36].

### 2.7. Congenital Stationary Night Blindness (CSNB)

CSNB, among all IRDs, is unique in being characterized by a non-progressive impairment of night vision and manageable fluctuations in visual acuity, with mutations in genes such as *RHO*, *GRM6*, and *TRPM1* that alter the function of rods or the signaling between photoreceptors and bipolar cells. Mutations in *RHO* typically lead to abnormalities in the rod response to light, while *GRM6* and *TRPM1* are involved in the signaling pathway of ON bipolar cells [37,38].

Patients with CSNB have difficulty adapting to low-light conditions, although daytime vision may remain relatively preserved. Unlike progressive IRDs, management of CSNB focuses on the patient’s existing vision. This includes strategies such as assistive lighting, low vision aids for dimly lit environments, and educational accommodations. Identifying the causative genetic mutation is essential for diagnosis, and while there is currently no curative treatment, understanding the genetic basis of CSNB is critical for potential future therapies [37,38].

### 2.8. Bestrophinopathies

Bestrophinopathies are a group of inherited retinal disorders caused by mutations in the *BEST1* gene, which encodes the bestrophin-1 protein. This protein primarily functions as a chloride ion channel in the RPE. The mutations can be either AD or AR, leading to various clinical manifestations, such as Best disease, characterized by a yellowish, evolving macular lesion sometimes referred to as “egg yolk,” and other forms such as AR bestrophinopathy, adult-onset vitelliform macular dystrophy, AD vitreoretinochoroidopathy, pattern dystrophy, and RP [39,40].

Currently, there are no definitive cures for bestrophinopathies, so clinical management focuses on monitoring disease progression and maintaining visual function through therapeutic and visual aids. Research is actively focused on developing gene therapies to correct the pathogenic mutations underlying these conditions, aiming to restore normal *BEST1* function in RPE cells to slow or halt disease progression [39,40].

## 3. Key Genes Involved in Syndromic Forms of IRDs (Figure 2)

### 3.1. Bardet–Biedl Syndrome (BBS)

BBS represents a complex, multisensory inherited retinal disorder that not only involves vision loss but also affects multiple organ systems. The clinical manifestations extend beyond vision impairment and include obesity, polydactyly, renal anomalies, and, at times, cognitive disturbances [41,42]. BBS is a disease with mutations identified in more than 20 genes such as *BBS1*-*BBS12* and others. These genes are involved in the function of primary cilia, cellular structures that play a key role in cell signaling pathways [42,43,44]. Recent studies have revealed an unexpected intersection of *BBS* genes with inflammatory signaling pathways. For example, research has indicated upregulation of Jak-STAT and NF-κB pathways, particularly in patients with *PKD1* mutations, which play a crucial role in regulating the immune response in polycystic kidney disease, a condition sometimes associated with BBS. These findings have shed light on new dimensions of BBS, suggesting that inflammation could be a contributing factor to disease progression. Such insights have prompted the study of immunomodulatory drugs as potential therapeutic options [43,44,45].

Given the clinical variability observed in BBS, personalized medicine approaches that consider the specific genetic composition and resulting pathophysiology are increasingly recognized as essential for effective management. Diagnosis is important, as medical therapy for obesity is available for BBS. Management of the multisystemic nature of BBS, requires a multidisciplinary approach focusing on vision preservation, treatment of systemic complications, and specific disease mutations to aid in genetic counseling and risk assessment. Due to the multiple comorbidities, multiple specialties are often required for appropriate medical management, such as genetics, nephrology, endocrinology, and ophthalmology. Moreover, early diagnosis may help prevent the development of obesity through the use of new medications, such as GLP-1 analogues and selective agonists of the MC4R receptor (setmelanotide). These drugs are licensed for weight loss, improved metabolic function, and cardiovascular outcomes in patients with type 2 diabetes.

### 3.2. Usher Syndrome

Usher syndrome is the most common genetic condition affecting both hearing and vision, classified into three main types, each of which involves a combination of hearing loss and RP, with varying severity depending on the type [46,47].

Usher type 1: This is the most severe form of Usher syndrome, characterized by congenital profound deafness, vestibular dysfunction, balance issues, and an early onset of symptoms of RP. The main genes involved in this type include the following:

○ *MYO7A (USH1B)*
○ *USH1C (Harmonin)*
○ *CDH23 (Cadherin-23)*
○ *PCDH15 (Protocadherin-15)*
○ *SANS (USH1G)*

These genes are essential for the function and structure of the sensory cells in the inner ear, as well as the photoreceptors in the retina. Mutations in these genes compromise both hearing and vision, leading to progressive sensory loss [47,48].

Usher type 2: It is characterized by moderate to severe hearing loss and the onset of RP symptoms occurring later, typically during adolescence or young adulthood. The main genes associated with this type include:

○ *USH2A (Usherin)*
○ *GPR98 (VLGR1)*
○ *DFNB31 (Whirlin)*

*Usherin* and *VLGR1* are involved in protein complexes that affect both the ear and the eye, and mutations in these genes lead to progressive sensory issues [49,50].

Usher type 3: This type is rarer and is characterized by progressive hearing loss, variable vestibular dysfunction, and symptoms of RP. The genes implicated include:

○ *CLRN1*
○ *HARS*

Mutations in these genes lead to progressive degeneration of both the photoreceptors and the sensory cells of the inner ear [50,51].

Each type of Usher syndrome has a specific profile of severity and disease progression, reflecting the functional diversity of the involved genes. These genes encode proteins that are crucial for the proper functioning of the sensory cells in the inner ear and the retina, both essential for hearing and vision. Mutations in these genes lead to a progressive loss of sensory functions, significantly impacting the patient’s quality of life. Early diagnosis is crucial for managing the syndrome. Timely intervention with assistive technologies, such as hearing aids or cochlear implants for deafness, and the use of visual aids for managing RP, are essential for improving quality of life and supporting the patient’s autonomy. Additionally, psychological support and genetic counseling are vital for addressing the multifactorial aspects and familial implications of the syndrome [46,47,48,49,50,51].

### 3.3. Familial Exudative Vitreoretinopathy (FEVR), Norrie Disease, and Pseudoglioma

FEVR is a genetic disorder that affects the development of retinal vasculature and can lead to vision loss through complications such as RD. Mutations in genes such as *NDP*, *FZD4*, *TSPAN12*, and *LRP5* disrupt the Wnt signaling pathway, a critical mechanism for the development and maintenance of retinal vessels. Treatment typically involves laser therapy or cryotherapy to address abnormal vascularization, and in some cases, surgical intervention may be required to correct RD [52,53]. Norrie disease is a rare X-linked disorder caused by mutations in the *NDP* gene. It primarily affects the eyes, leading to blindness, but can also result in hearing loss and cognitive disabilities. A characteristic finding of Norrie disease is the presence of a fibrous mass behind the ocular lens, resembling a pseudoglioma [52,53,54]. Management is supportive and focuses on visual rehabilitation and hearing services when possible. The complexity of these disorders highlights the critical role of genetic pathways in ocular development and underscores the importance of precision medicine for diagnosis and treatment.

### 3.4. Ocular Disorders Associated with PAX6

Ocular disorders related to PAX6 encompass a wide range of anomalies caused by mutations in the *PAX6* gene, which plays an essential role in ocular development. Mutations can lead to conditions such as aniridia, keratitis, cataracts, glaucoma, RP, and other ocular abnormalities. In some cases, *PAX6* mutations can also affect brain development, resulting in neurological implications. Management of *PAX6*-related disorders is personalized based on the specific ocular anomalies present and often involves coordinated care among ophthalmologists, genetic counselors, and neurologists [55,56].

### 3.5. Juvenile Neuronal Ceroid Lipofuscinosis (JNCL) or Batten Disease

JNCL, commonly caused by mutations in the *CLN3* gene, is a type of neuronal ceroid lipofuscinosis that typically presents in childhood with progressive vision loss followed by neurological decline. The gene is involved in lysosomal function, and its mutations lead to the accumulation of lipofuscin within lysosomes, disrupting cellular function. Patients may initially present to the ophthalmologist with vision loss before other symptoms become apparent. As the disease progresses, seizures, motor decline, and cognitive deterioration develop. Treatment is supportive and palliative, focused on symptom management and maintaining quality of life. Exploration of enzyme replacement therapy and gene therapy offers hope for future treatments [57,58].

### 3.6. Mitochondrial Eye Diseases

Mitochondrial eye diseases, which include a range of optic neuropathies and IRDs, have increasingly been attributed to mutations in both mitochondrial and nuclear genes (mtDNA). Involving over 40 genes, these conditions reflect their genetic diversity and particularly manifest in organs with high energy demands, such as the eye [59]. They can lead to optic neuropathy, retinal degeneration, or other defects in the visual pathways. Mitochondrial disorders involve dysfunction in mitochondrial energy production, oxidative stress, mitochondrial DNA instability, abnormalities in mitochondrial dynamics and quality control, as well as disturbances in interorganelle communication. The emphasis on Whole Genome Sequencing (WGS) in mitochondrial disease research, including in ocular pathologies, has highlighted its potential to uncover hidden inheritance and enable better understanding and treatment of these complex conditions, such as Leber hereditary optic neuropathy (LHON) or AD optic atrophy Kjer type (ADOA) [59,60,61]. LHON is a maternally inherited disease caused by mutations in mtDNA respiratory chain complex I subunit genes: *MT-ND1*, *MT-ND4*, and *MT-ND6*; extremely rare is AR inheritance for *NDUFS2* in LHON-like phenotype. ADOA is caused by mutations of the mtDNA in the gene *OPA1* or other genes involved in *OPA* in isolated or syndromic forms. Antioxidant therapies and gene therapies are emerging as promising approaches to treat ophthalmic manifestations of mitochondrial diseases, with the goal of optimizing mitochondrial function, preserving retinal function, and thereby maintaining visual capacity in patients [60,61].

### 3.7. Screening of High Myopia Syndromic Monogenic (msHM)

High myopia syndromic monogenic (msHM) represents a group of conditions where high myopia is associated with systemic features due to mutations in a single gene. Advances in WES, supported by initiatives such as the Myopia Associated Genetics and Intervention Consortium (MAGIC), have led to the development of targeted panels for genes associated with msHM [62,63]. This screening has identified critical mutations in multiple genes, linking them to over 85 distinct ocular diseases. This genetic exploration has been particularly significant for retinal dystrophies, connective tissue disorders, and malformations of the cornea and lens. For example, pathogenic variants in genes such as *TRPM1*, *CACNA1F*, *FZD4*, and others have been demonstrated to be responsible for msHM. The screening underscores the value of molecular subtyping for precise genetic counseling and for guiding multidisciplinary care, which is essential for managing the broad range of phenotypes presented by msHM [62,63].

## 4. Clinical Practice Implications, Criteria, and Diagnostic Techniques Applied to IRDs

Clinical studies illustrate the complex interaction between mutations and clinical manifestations, highlighting the nuances of diagnosis, management, and therapeutic interventions. They emphasize the diversity and complexity of the genetic and phenotypic manifestations of IRDs through the following points [5,8,64]:

Complex diagnostic pathways: The diagnostic pathway for each patient may involve multiple clinical evaluations and genetic tests, underscoring the need for a multidisciplinary approach.

Importance of longitudinal follow-up: Continuous monitoring and reassessment may lead to revisions of the diagnosis and management plan, reflecting the evolving understanding of the genetic contributions to IRDs.

Potential for targeted therapies: The identification of specific mutations opens the door to personalized medicine, including potential participation in gene therapy studies, which are increasingly becoming a reality in the management of these complex disorders.

Cases encountered in everyday medical practice not only provide valuable insights for clinicians and researchers, but also highlight the importance of detailed genetic analysis in shaping the therapeutic landscape of IRDs. A detailed exploration serves as a resource for understanding the multifaceted nature of these conditions and paves the way for future advances in diagnosis and treatment approaches.

In this context, the diagnostic pathway of IRDs has seen significant advancements that have transformed criteria, introduced innovative technologies, and optimized clinical practices for greater accuracy and timeliness in diagnosis [5,8,64,65,66]. The integration of cutting-edge genetic data with state-of-the-art imaging technologies has allowed for more precise assessments. With an improved understanding of these diseases, diagnostic criteria have evolved toward more structured evaluations supported by technology. The introduction of ERG in the late twentieth century revolutionized the field, allowing the measurement of electrical responses from rod and cone photoreceptors, thus identifying various subtypes of IRDs through the analysis of a- and b-waves, which reflect the activity of photoreceptor cells, bipolar cells, and Müller cells.

Today, the clinical diagnosis of IRDs combines a detailed patient history with in-depth ocular examinations, such as those described below [64,65,66,67,68,69]:

Visual field (VF): An essential psychophysical test for assessing useful visual field perception and its loss, which is repeated at regular intervals.

Optical coherence tomography (OCT) and OCT angiography (OCTA): A noninvasive imaging technique that provides high-resolution images of the choroid, retina, and optic disc structures, enabling, moreover, the detection of morphological and vascular changes in blood flow with high-precision follow-up

Retinography: An imaging test used to compare the appearance of the ocular fundus over time with color photographs.

Fundus autofluorescence (FAF): An imaging test useful for identifying the accumulation of lipofuscin or areas of atrophy in the RPE due to excess lipofuscin.

Fluorescein angiography (FA) or indocyanine green angiography (ICG): Exams performed with contrast agents to detect in detail pathologies or secondary complications such as chorioretinal neovascularization.

These diagnostic tools, alongside clinical expertise and genetic analysis, offer a comprehensive approach to the management of IRDs, improving the accuracy and timeliness of diagnoses and enabling better patient care.

## 5. Genetic Analysis Technologies: From WES to Advanced Genomic Profiling

**Figure 4:**
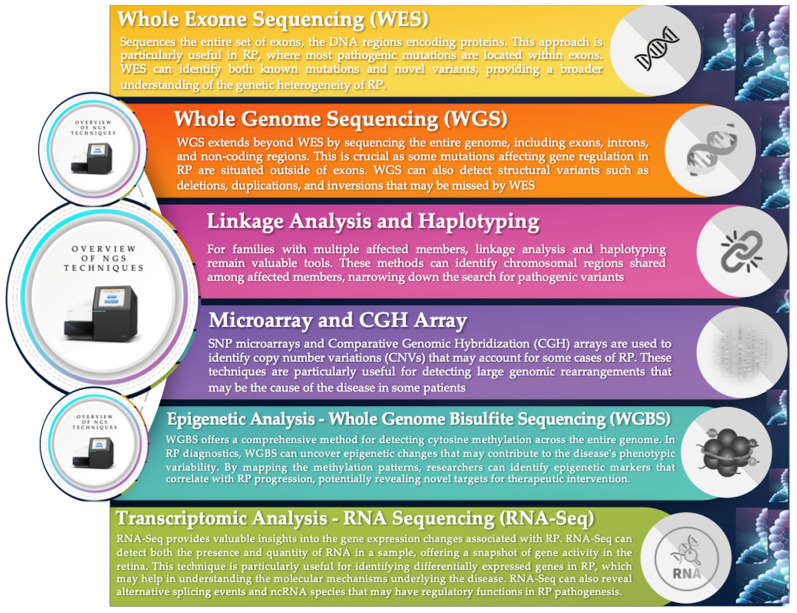
Overview of the main next-generation sequencing (NGS) analysis methods to support genetic investigations of inherited retinal dystrophies (IRDs). From whole exome sequencing (WES) to modern transcriptomic and epigenomic techniques, these are cutting-edge techniques and technologies that can provide a decisive contribution to translational research and diagnostics of IRD.

IRDs benefit from modern genetic sequencing techniques and analysis, enabling precise diagnosis and the identification of responsible mutations. The main technologies used are (Figure 4) [70,71,72,73,74,75,76,77,78,79,80,81,82]:

Whole exome sequencing: WES sequences all exons, the regions of DNA that encode proteins, where pathogenic mutations are commonly found. This method not only confirms already known mutations but also discovers new variants, contributing to a better understanding of the complex genetic heterogeneity in IRDs. Recent innovations in WES have expanded our ability to map these variations at a more detailed and accurate level [70,71,72].

Whole genome sequencing: WGS extends the analysis beyond exons to include introns and non-coding regions of DNA, which are crucial for better understanding mutations that affect gene regulation and are not detectable by WES. This approach can identify structural variants such as deletions, duplications, and inversions, providing a comprehensive view of an individual’s genetic landscape [73,74,75,76].

Linkage and haplotyping analysis: Linkage analysis helps identify chromosomal regions that are transmitted together with a specific disease within a family. This method is especially useful in large families in which the exact mutation is unclear. By combining this information with haplotyping, it is possible to narrow down the search for pathogenic variants [73,74,75,76,77].

Microarray and comparative genomic hybridization (CGH) arrays: Microarrays and CGH arrays are effective in identifying copy number variations in DNA that could be the cause of IRDs in some patients, revealing large genomic rearrangements that may include critical deletions or duplications. These tools are particularly useful for detecting genomic anomalies that may not be visible with other sequencing techniques. The effectiveness of these approaches in revealing variants that cause IRDs has been demonstrated in several studies, where the results have significantly improved diagnosis and clinical interpretation of IRDs [74,75].

Epigenetic analysis—whole genome bisulfite sequencing (WGBS): WGBS analyzes cytosine methylation, providing a complete map of epigenetic patterns that may influence gene expression and the disease phenotype. The information obtained is crucial for understanding how epigenetic modifications may contribute to the clinical manifestations of IRDs and for identifying new targets for targeted therapeutic interventions [74,75,76].

Transcriptomic analysis—RNA sequencing (RNA-Seq): RNA-Seq provides a detailed overview of gene activity by analyzing the presence and quantity of RNA in the sample. This technique is particularly valuable for identifying genes with differential expression in pathological conditions. RNA-Seq can also reveal alternative splicing events and the presence of non-coding RNAs, which may play critical regulatory roles in the progression of IRDs [76,77,78].

The integration of genetic data with specialized clinical examination and innovative instrumental techniques for follow-up represents a significant advancement in IRD diagnostics. This approach allows for greater diagnostic accuracy since the phenotypic manifestation of these mutations can vary greatly between individuals. It is also possible to predict disease progression, which can sometimes be faster, and integrate this with personalized medicine according to the specific stage of the patient’s disease, up to the point of identifying and selecting candidates for emerging therapies, such as gene therapy [79,80,81,82].

It is the synergy between advanced genomic profiling and precision imaging diagnostics that enables physicians to make informed decisions, offering patients targeted treatment strategies and the hope of achieving better outcomes.

## 6. Effective Patient Communication: Genetic Counseling, Interpretation of Reports, and Management Strategies for IRDs

The interpretation of genetic reports is a cornerstone of genetic counseling. Doctors and genetic counselors must translate complex genetic data into terms that patients can understand. The classification of genetic variants, based on the criteria from the American College of Medical Genetics and Genomics (ACMG), guides the interpretation of the reports, depending on the applied criteria [83]. This system classifies variants into five tiers:

❖ Pathogenic (P)
❖ Likely pathogenic (LP)
❖ Uncertain significance (VUS)
❖ Likely benign (LB)
❖ Benign (B)

It is essential that patients and families understand the meaning of these results, specifically, which variants may influence the disease and which are benign polymorphisms. Knowing the specific genetic mutation can help predict disease progression, tailor surveillance and management strategies, and guide eligibility for new treatments such as gene therapy [5,84]. Genetic testing can identify not only pathogenic variants but also deep intronic variants and copy number variations, expanding the scope of potential therapeutic targets.

Moreover, genetic counseling for IRDs often includes discussions about family planning, focusing on inheritance patterns and implications for current and future children. At-risk couples may consider advanced reproductive options such as in vitro fertilization with preimplantation genetic diagnosis (PGD), an approach that allows for the identification of embryos without the genetic mutations responsible for IRDs before implantation [85,86,87]. This technology is crucial for reducing the risk of transmitting genetic diseases to offspring. Counseling provides essential guidance on how certain hereditary conditions can be passed on to children. For instance, up to 4% of families with IRDs exhibit the coexistence of mutations in multiple disease-causing genes, complicating both reproductive and therapeutic counseling. Specialists provide detailed information on the likelihood of transmission of various mutations and options to mitigate the risk.

PGD is used to genetically analyze embryos before implantation, ensuring that only those without known genetic mutations are selected for pregnancy. This technique is an integral part of modern strategies for managing IRDs, offering families preventive options before conception. The support provided by genetic counselors is vital in helping couples understand the implications of reproductive decisions, especially when considering advanced technologies such as PGD. It is crucial to discuss the likelihood of success and the ethical challenges related to the use of these technologies, providing a comprehensive framework to help couples make informed decisions [85,86,87].

In any case, the genetic counselor plays a critical role in providing the necessary support and information to help families navigate the complex landscape of IRDs. These scenarios demonstrate the breadth of genetic counseling, which not only concerns the transmission and implications of genetic disorders but also assists in making decisions regarding the management of current and future health. As the field of genetics continues to progress, genetic counselors are key facilitators in translating these complexities into actionable and understandable information for patients and their families.

## 7. Focus on Gene Therapy for IRDs: Genome Editing, Ethical and Regulatory Issues

Gene therapy represents a revolutionary frontier in the treatment of IRDs, offering new hope for conditions previously considered incurable. Developed in the late 1980s, it is performed by manipulating gene expression to treat hereditary diseases, using cloning and sequencing of altered or missing genes. Thanks to recombinant DNA techniques, the genetic makeup of inactivated microbial vectors is specifically replaced with a healthy gene, allowing the replacement of defective sequences in patient cells, so that the cell synthesizes the correct proteins [88,89,90]. The use of viral vectors, especially adeno-associated viruses (AAVs), is essential to deliver correct copies of genes to retinal cells. An emblematic example of success in this field is Luxturna (voretigene neparvovec), the first gene therapy approved by the FDA to treat retinal dystrophy associated with biallelic mutations in the *RPE65* gene. This treatment has demonstrated the potential for visual recovery offered by gene therapy with a well-defined genetic basis. Other clinical studies on various IRDs, such as choroideremia caused by mutations in the CHM gene, have reported significant improvements in visual acuity and retinal sensitivity, using AAV2-REP1 administered under the retina, indicating a promising therapeutic path for this condition as well. However, variability in results across different studies and dosages highlights the complexity of defining effective treatment protocols and measures for the therapies (Table 1) [88,89,90].

The next step that researchers are focusing on is “Genome Editing”. This is a branch of high precision genetic engineering that aims to delete, modify, replace, or insert DNA at specific sites, rather than randomly, as has been done until now. Engineered nucleases in the form of “molecular scissors,” which break the double-stranded DNA at a specific site, are used for this purpose. The DNA is then forced to repair itself through homologous recombination [91,92,93].

Genome editing technologies, such as those using nucleases called CRISPR-Cas, have opened new possibilities for directly correcting genetic defects at the DNA level and potentially curing genetic diseases. For example, for X-linked retinoschisis, caused by mutations in the *RS1* gene, gene therapy approaches have shown improvements in retinal structure and function in animal models, and clinical trials are underway, already providing promising initial results. Similarly, for Stargardt disease, the most common inherited macular dystrophy, gene therapies are in progress to manage lipofuscin accumulation and prevent further degeneration of the RPE and, consequently, photoreceptors [93,94,95,96].

These cases demonstrate how the integration of new gene therapies could transform the treatment of IRDs, leading to new therapeutic possibilities and significantly improving the quality of life for patients. The scientific community has made significant progress in improving the efficiency, precision, and safety of genome editing tools. At this point, however, the controversial chapter has opened regarding the ethical discussions surrounding informed consent, potential off target effects, regulations on immediate adverse consequences, and the long-term implications of genome editing on individuals and populations. Nonetheless, genome editing is becoming a crucial component in the treatment strategy for IRDs, offering targeted and personalized interventions, still largely to be explored [95,96,97].

Moreover, the challenges that current gene therapies for IRDs face include the genetic complexity of these diseases, which may involve multiple mutations in several genes. The methods of gene therapy delivery, such as subretinal or intravitreal injections, carry both risks and benefits. The invasiveness of subretinal injections, necessary to reach the photoreceptors, must be balanced with the potential immune responses and the systemic spread associated with intravitreal injections, while also being aware that these technologies are still relatively new. Ongoing research is essential to improve the effectiveness and safety of therapies with personalized approaches for the patient, given the heterogeneity of IRDs. The success of gene therapies in clinical trials and their approval by regulatory agencies such as the FDA herald a new era in the treatment of IRDs, shifting from symptom management to the possibility of restoring vision while ensuring safety and efficacy. Advanced therapies, such as gene therapy and genome editing, present unique risks and benefits that must be carefully balanced. The complexity of the data and ethical considerations require a rigorous evaluation process to ensure that proposed treatments are both safe and effective [96,97,98].

Furthermore, the ethical landscape of genetic testing for IRDs in pediatric patients is rich with nuances. Considerations involve the timing and scope of genetic testing, especially in children, where consent and the potential future impacts must be carefully weighed. The American Academy of Pediatrics emphasizes the necessity of informed consent and assent in pediatric practice, supporting the need for diagnostic testing when effective measures are available to prevent, treat, or improve conditions. The approach to testing should be cautious and, if possible, delayed until the child can participate in the decision-making process [99,100,101,102].

However, children with IRDs may encounter significant difficulties in social integration and education due to their visual limitations. It is essential that they have access to appropriate educational resources and support systems to ensure full participation in social and school life. Genetic counseling and healthcare professionals play a crucial role in providing useful information, necessary services, and social integration. Issues such as privacy, potential discrimination, and the psychological impact of genetic information remain central to the discussion [99,100,101,102].

### Clinical Results and Long-Term Effects of the Luxturna Gene Therapy

Luxturna is the first gene therapy approved by the FDA for the treatment of RP associated with biallelic mutations in the *RPE65* gene, one of the causes of hereditary congenital blindness. Luxturna is an innovative treatment that involves injecting healthy copies of the *RPE65* gene directly into the patient’s retina using an AAV2 vector [90,91,103]. In early clinical studies, patients treated with Luxturna showed significant improvements in low light vision. In a phase 3 trial, the treatment resulted in improvements in visual acuity measured by the light sensitivity test, with an average improvement of about 2 lines on the early treatment diabetic retinopathy study grid (ETDRS) compared to untreated patients. The treated patients also showed improvements in contrast sensitivity performance, and they continued to improve or maintain their vision 1–2 years after treatment [91,103,104].

A 3-year follow-up showed that most patients maintained or even improved their ability to adapt to low light conditions, one of the most critical aspects for patients with *RPE65* mutations (Table 2).

Although the results have been positive for many patients, it was observed that the effectiveness of the treatment can vary. Some patients showed less significant improvements, likely due to factors such as age at the time of treatment and the degree of pre-existing retinal degeneration. Moreover, visual improvements had a significant impact on the patients’ quality of life, enhancing their ability to perform daily activities such as reading, driving, and social interaction [91,103,104,105].

However, it should be remembered that, in some cases, gene therapy can also have negative side effects, e.g., the progression of atrophy, as seen with Luxturna or in STGD therapy trials. Long-term follow-up will be important to define treatment success.

In addition to Luxturna, other AAV-based gene therapies are being tested to treat different inherited retinal diseases, such as choroideremia and X-linked retinoschisis.

Choroideremia, caused by mutations in the *CHM* gene, is another example where AAV gene therapy has shown promising results. In a clinical study, patients treated with the AAV2-REP1 vector, which provides a healthy copy of the *CHM* gene, showed improvements in retinal sensitivity and visual acuity. In a phase 3 clinical trial, after subretinal treatment, patients showed a significant increase in retinal sensitivity compared to the control group. The improvements were particularly evident in patients who still had good residual retinal function prior to treatment [106,107].

In a trial on X-linked retinoschisis, caused by mutations in the *RS1* gene, improvements in retinal morphology and visual function were observed in animal models and some treated patients [106,107].

The clinical results of AAV-based gene therapies for treating IRDs are encouraging, but the long-term effectiveness and variability of results highlight the need for further research. The use of gene therapies, such as Luxturna, has already led to significant visual improvements, especially in patients with early diagnosis and less retinal damage. However, these therapies are not without risks and limitations, such as the need for subretinal injections and the possibility of immune reactions or long-term side effects [108,109].

Advancements in genome editing technologies like CRISPR-Cas9, which offer the potential to directly correct genetic defects, could lead to even more precise and lasting treatments in the future. However, further research is needed to optimize the safety and effectiveness of these techniques [108,109].

## 8. Future Directions in Research and Therapy for IRDs

Innovative studies on IRDs are rapidly transforming the landscape of potential treatments. Ongoing research is not limited to studying gene integration therapy but also explores the capabilities of genome editing strategies, particularly CRISPR-Cas9 [109,110]. These advancements suggest that while gene integration may be effective for RA and X-linked forms caused by loss-of-function mutations, it may not be ideal for DA forms with gain-of-function mutations. For this reason, gene integration therapy is being adapted to address these variations, with alternatives such as lentiviruses and non-viral vectors like plasmids and DNA nanoparticles being considered for their ability to carry larger genes, which are otherwise inaccessible to AAV-mediated approaches. Genome editing techniques like CRISPR-Cas9 offer the promise of a permanent cure for a range of mutations through targeted genomic modifications [110,111].

Other future research directions involve artificial intelligence (AI) and machine learning (ML), which are significantly improving the interpretation of complex genetic data and the identification of phenotypic patterns associated with genetic variations [112,113]. These technologies are essential in the development of personalized medicine strategies and are increasing the accuracy and efficiency of diagnoses and treatment plans. AI and ML have proven particularly useful in analyzing images obtained through OCT, FAF, and retinography, enabling the segmentation and recognition of pathological features with high precision [111,112,113]. A recent study highlighted the use of AI in managing inherited retinal disorders, from diagnosis to treatment, showcasing the major advantages and challenges of using such methods [112,113].

In this context, international cooperation and the creation of global registries, such as the Global Registry or the IRD Registry, or similar programs that collect data worldwide, are essential for accelerating research and developing therapies [114]. These initiatives facilitate the sharing of data, resources, and knowledge on a global scale, enhancing the effectiveness of clinical studies and guiding research toward new therapeutic approaches. Patient registries are crucial for rare IRDs, as they provide valuable data that can be compared globally, helping inform and guide clinical decisions and future research (Table 3). The next decade promises significant progress in the management of pediatric IRDs [113,114,115].

It is expected that ongoing discoveries will lead to the introduction of numerous gene therapy products, the refinement of CRISPR-based therapies, and the increasing integration of AI and ML into research and clinical practice. These developments, combined with increased international collaboration, should usher in an era where personalized therapies, based on individual genetic profiles, become the norm, significantly improving outcomes for affected subjects. The shift will bring more effective information and improved quality of life for patients and their families, globally transforming the management and treatment of inherited retinal diseases [113,114,115].

## 9. Conclusions

In conclusion, it can be affirmed that initiating a global commitment to improve access to services and genetic therapies for IRDs is of fundamental importance. Clinical studies and therapeutic strategies are rapidly expanding, but access to care for IRDs varies greatly. Some countries have advanced infrastructures that support genetic testing and innovative therapies, while others lack the necessary resources to provide these services.

Ethical guidelines must balance the potential benefits of early diagnosis and intervention with the risks and implications of genetic knowledge. Regulatory challenges involve ensuring safe and effective treatments, while global disparities in access to care require concerted efforts to bridge the gap. These issues underscore the importance of a multidisciplinary approach to addressing the needs of IRD patients and their families. Social considerations include ensuring that children with IRDs are supported in their educational and social endeavors.

Over the last three decades, more than 300 genes responsible for IRDs have been identified. This genetic clarification has facilitated the advent of precise and targeted diagnostic and therapeutic interventions for specific genetic etiologies. With current approaches to genetic testing reaching a mutation identification rate of 60–80%, the promise of a genetic diagnosis for all affected individuals is nearly within reach. Technological advancements are poised to expand genetic testing, and with the anticipated inclusion of standardized genetic and phenotypic information in global open databases, the interpretation of genetic variants is expected to become increasingly accurate.

The approval of the first gene therapy for IRDs by regulatory agencies such as the FDA has paved the way for further innovations, including trials for the enhancement of genes for recessive loss-of-function mutations and the ablation and replacement of genes for dominant diseases. Other promising approaches, such as stem-cell-derived RPE for Stargardt disease and retinal progenitor cells for RP, highlight the dynamic landscape of research. Additionally, efforts to develop mutation-independent therapies aimed at shared pathogenic mechanisms offer hope for treating broader segments of the IRD population. However, clinical studies are complex endeavors that require balancing the interests of patients, society, and companies. As more resources are funneled into this field, it is essential to prioritize studies based on pathology and principles that foster valid, safe, and rapid outcomes.

Looking to the future, there is great optimism that the integration of AI and ML will improve the landscape of personalized treatments, advancing the promise of precision medicine and better outcomes for patients. A collective call is needed to sustain this momentum, support the translation of research into clinical practice, and commit to equitable care for all affected individuals. The vision for the next decade is to provide innovation, collaboration, and, most importantly, ongoing commitment to improving the lives of people with visual impairment or risk of blindness.

## Figures and Tables

**Table 1:** Table showing some interventional clinical trials (CT) with phase, study type, gene targeted, disease phenotype, administration drug via intravitreal (IVT) or subretinal (SR) injection, patient cohort, and available links.

CT/Phase	Study Type	Gene	Disease	Drug	Cohort	Link
NCT05392179 Phase 2	Open label non-randomized	Rhodopsin mutations including P23H	Retinitis pigmentosa	ADX-2191 serial IVT (*methotrexate*)	**8**	https://clinicaltrials.gov/study/NCT05392179?term=retinitis%20pigmentosa&rank=3#study-plan (accessed on 11 May 2025)
NCT03252847 Phase 1/2	Open label multicenter, dose escalation trial	RPGR	X-linked retinitis pigmentosa	AAV5-RPGR vector SR	49	https://clinicaltrials.gov/study/NCT03252847?term=NCT03252847&rank=1 (accessed on 11 May 2025)
NCT02317887 Phase 1/2a	Prospective, single-center dose escalation	RS1	Retinoschisis X-linked	AAV-RS1 vector IVT	12	https://clinicaltrials.gov/study/NCT02317887?tab=results (accessed on 11 May 2025)
NCT03140969 Phase 1/2	Open label, multiple dose escalation	CEP290 p.Cys998X mutation	Amaurosi congenita di Leber	QR-110 RNA antisense oligonucleotide targeting the c.2991+1655A>G IVT	11	https://clinicaltrials.gov/study/NCT03140969 (accessed on 11 May 2025)
NCT05417126 Phase 2a	Multicenter open label	ABCA4, ELOVL4, PROM 1	Stargardt disease	vMCO-010 vector IVT	6	https://clinicaltrials.gov/study/NCT05417126?term=NCT05417126&rank=1 (accessed on 11 May 2025)
NCT05748873 Phase 1/2A	Multicenter double masked randomized	RHO, PDE6A, PDE6B	Rod cone dystrophies	SPVN06 vector SR	33	https://clinicaltrials.gov/study/NCT05748873?term=NCT05748873&rank=1 (accessed on 11 May 2025)
NCT03758404 Phase 1/2	Open label Multicenter	CNGA3	Achromatopsia	AAV-CNGA3 vector SR	11	https://clinicaltrials.gov/study/NCT03758404?term=NCT03758404&rank=1 (accessed on 11 May 2025)

**Table 2:** Gene therapies and clinical results for Luxturna and other gene therapies with adenoviral vectors used (AAVs).

**Therapy**	**Disease Treated**	**Gene**	**AAV**	**Clinical Outcome**
Luxturna	*RPE65*-related retinal Dystrophy	*RPE65*	AAV2	Improvement in low-light vision, long-term visual maintenance
Gene therapy for choroideremia	Choroideremia	*CHM*	AAV2-REP1	Increased retinal sensitivity, improved visual acuity
Gene therapy for X-linked retinoschisis	X-linked retinoschisis	*RS1*	AAV	Improvements in retinal morphology and visual acuity preliminary results
**Clinical Results, Follow-Up Duration and Significant Improvement**				
**Study/Trial**	**Follow-Up**	**Visual Outcome**	**Improvement**	**Observations**
Phase 3 Luxturna trial	1–2 years	Improvement in low light vision	About 2 lines on the ETDRS* grid	Variability in improvements among patients, impact on quality of life
3-year follow-up of Luxturna	3 years	Maintenance or improvement in vision	Better adaptation to low light conditions	Stable long-term results for most patients
Choroideremia AAV2-REP1 study	1 year	Increased retinal sensitivity	Improved sensitivity in the treated group	More noticeable improvements in patients with residual retinal function prior to treatment

* ETDRS: early treatment diabetic retinopathy study.

**Table 3:** Gene therapy efficacy relative to clinical variables in inherited retinal dystrophies (IRDs). Global initiatives and international cooperation.

**Clinical Variable**	**Patients with Significant** **Improvements**	**Patients with Lesser** **Improvements**	**Factors Associated with** **Reduced Improvement**
Age at treatment	Greater improvement in younger patients	Less improvement in older patients	Advanced age may affect response to treatment
Pre-existing retinal degeneration	Better results in patients with mild to moderate degeneration	Less pronounced results in patients with advanced degeneration	Presence of pre-existing retinal damage may limit treatment efficacy
**Global Initiatives and International Cooperation**			
**Initiative/Program**	**Primary Objective**	**Participants/Collaborators**	**Expected Benefits**
Global registry for IRDs	Collection of global data on inherited retinal diseases	Hospitals, clinics, and international research institutes	Accelerating research, international data comparison, development of personalized therapies
Single-country IRD records to compare	Creation of a global registry for IRD patients	Patient organizations, universities, research centers	Improving clinical decisions and therapeutic protocols

## Data Availability

Not applicable.

## Abbreviations

The following abbreviations are used in this manuscript:

AAVs	Adeno-associated viruses
ACMG	American College of Medical Genetics and Genomics
AD	Autosomal dominant
ADOA	Autosomic dominant optic atrophy Kjer type
AI	Artificial intelligence
AR	Autosomal recessive
B	Benign
BBS	Bardet–Biedl syndrome
CGH	Comparative genomic hybridization
CRDs	Cone and rod dystrophies
CSNB	Congenital stationary night blindness
ERG	Electroretinogram
ETDRS	Early treatment diabetic retinopathy study grid
FA	Fluorescein angiography
FAF	Fundus autofluorescence
FDA	Food and Drug Administration
FEVR	Familial exudative vitreoretinopathy
GWASs	Genome-wide association studies
ICG	Indocyanine green angiography
IRDs	Inherited retinal dystrophies
JNCL	Juvenile neuronal ceroid lipofuscinosis
LB	Likely benign
LCA	Leber congenital amaurosis
LHON	Leber hereditary optic neuropathy
LP	Likely pathogenic
MAGIC	Myopia Associated Genetics and Intervention Consortium
ML	Machine learning
msHM	High myopia syndromic monogenic
mtDNA	Mitochondrial and nuclear genes
NGS	Next-generation sequencing
OCT	Optical coherence tomography
OCTA	Optical coherence tomography angiography
P	Pathogenic
PGD	Preimplantation genetic diagnosis
RD	Retinal detachment
RNA-Seq	RNA sequencing
RP	Retinitis pigmentosa
RPE	Retinal pigment epithelial
STGD	Stargardt disease
VF	Visual field
VUS	Uncertain significance
WES	Whole exome sequencing
WGBS	Whole genome bisulfite sequencing
WGS	Whole genome sequencing
XLRS	X-linked retinoschisis
